# Supplementary material for: Experience of living with chronic pain in conjunction with surgery for ulnar nerve entrapment at the elbow—A qualitative study
Source: PLoS One. 2024 Jun 28;19(6):e0306327. doi: 10.1371/journal.pone.0306327 (PMC11213347; doi:10.1371/journal.pone.0306327)
Supplement: S1 File — (PDF) [file pone.0306327.s001.pdf]

The semi-structured interview guide

Opening questions

Start with some opening questions

- How old are you?
- Which arm has been surgically treated?
- Are you right handed or left handed?
- How long has it been since the surgery?
- What's your occupation?
- Do you have a family?
- What do you do in your spare time?
- Do you do any sports or have other training activities?

Preoperative difficulties/symptoms?

- Tell me what made you seek medical care for your arm?
- Describe in detail, what symptoms you had preoperatively, concerning your arm?
- Describe your ability to perform daily activities such as cooking, grocery shopping, dressing etc?
- Describe how your working life was affected?
- Describe how your family life and/or social life was affected?
- Describe, in as much detail as possible, how your mental health was affected?
- Describe how your sleep was affected?
- Describe how your hobbies were affected?

… how did you handle that?

Postoperative difficulties/symptoms and adaptive strategies

- Can you describe in as much detail as possible, what difficulties or symptoms you have today?
- In what ways are the symptoms/difficulties different today compared to before the surgery?
- In what way did the surgery affect your:
    - ability to perform daily activities?
    - work life?
    - family and social life?
    - mental health?
    - sleep?
    - leisure activities?

… how did you handle that?

Consequences and adaptive strategies

- What other kinds of strategies or solutions have you used to deal with your problems? What alleviates or makes your problems easier to deal with?
- Describe the ways in which the situation with your arm means that you live your life differently from what you would have liked to do.
- What would you like to say to someone with similar conditions facing similar surgery?
- Is there anything you would like to add concerning consequences or adaptive strategies that we have not talked about?
