# Supplementary material for: Experience of living with chronic pain in conjunction with surgery for ulnar nerve entrapment at the elbow—A qualitative study
Source: PLoS One. 2024 Jun 28;19(6):e0306327. doi: 10.1371/journal.pone.0306327 (PMC11213347; doi:10.1371/journal.pone.0306327)
Supplement: Attachment — Submitted filename: Response to reviewers.docx [file pone.0306327.s002.docx]

RESPONSE TO REVIEWERS - rebuttal letter

Dear Editor,

Thank you for your mail concerning our manuscript entitled ”Experience of living with chronic pain in conjunction with surgery for ulnar nerve entrapment at the elbow – a qualitative study”. We appreciate the comments made by the reviewers and we have performed the requested changes in the manuscript.

A marked-up copy of our manuscript that highlights the performed changes made to the original version is attached in the system (labeled as 'Revised Manuscript with Track Changes'.

Furthermore, an unmarked version of our revised paper without tracked changes as uploaded in the system and labeled as 'Manuscript'.

**Question:** We note that the grant information you provided in the ‘Funding Information’ and ‘Financial Disclosure’ sections do not match.

**Reply:** We have no disclosures related to the present manuscript. However, our universities and hospitals have received research grants for performing research ass the present one.

**Question**: When you resubmit, please ensure that you provide the correct grant numbers for the awards you received for your study in the ‘Funding Information’ section.

**Reply:** Updated statement added in the cover letter.

**Question:** We note that you have indicated that there are restrictions to data sharing for this study. PLOS only allows data to be available upon request if there are legal or ethical restrictions on sharing data publicly. For more information on unacceptable data access restrictions, please see <http://journals.plos.org/plosone/s/data-availability#loc-unacceptable-data-access-restrictions>.

If there are ethical or legal restrictions on sharing a de-identified data set, please explain them in detail (e.g., data contain potentially identifying or sensitive patient information, data are owned by a third-party organization, etc.) and who has imposed them (e.g., a Research Ethics Committee or Institutional Review Board, etc.). Please also provide contact information for a data access committee, ethics committee, or other institutional body to w

**Reply:** An additional paragraph is added concerning this issue. See below.

Reviewer's Responses to Questions

**Comments to the Author**

**Question:** Is the manuscript technically sound, and do the data support the conclusions?

The manuscript must describe a technically sound piece of scientific research with data that supports the conclusions. Experiments must have been conducted rigorously, with appropriate controls, replication, and sample sizes. The conclusions must be drawn appropriately based on the data presented.

Reviewer #1: Yes

Reviewer #2: Partly

**Reply:** No comment required.

**Question:** Has the statistical analysis been performed appropriately and rigorously?

Reviewer #1: Yes

Reviewer #2: N/A

**Reply:** No comment required.

**Question:** Have the authors made all data underlying the findings in their manuscript fully available?

The [PLOS Data policy](http://www.plosone.org/static/policies.action#sharing) requires authors to make all data underlying the findings described in their manuscript fully available without restriction, with rare exception (please refer to the Data Availability Statement in the manuscript PDF file). The data should be provided as part of the manuscript or its supporting information, or deposited to a public repository. For example, in addition to summary statistics, the data points behind means, medians and variance measures should be available. If there are restrictions on publicly sharing data—e.g. participant privacy or use of data from a third party—those must be specified.

Reviewer #1: No

Reviewer #2: Yes

**Reply:** We have included a separate comment concerning this issue based on the approval by our Ethical committee:

***“Data availability statement***
*Public access to research data on humans is restricted by the Swedish Authorities (Public Access to Information and Secrecy Act;* [*https://www.government.se/information-material/2009/09/public-access-to-information-and-secrecy-act/*](https://www.government.se/information-material/2009/09/public-access-to-information-and-secrecy-act/)*), but data can be made available for researchers after a special review that includes approval of the research project by both the Swedish Ethical Review Authority (*[*https://etikprovningsmyndigheten.se/en/*](https://etikprovningsmyndigheten.se/en/)*) and the local authorities' data safety committee in the health care sector in Region Skåne, Sweden;* [*https://vardgivare.skane.se/kompetens-utveckling/forskning-inom-region-skane/utlamnande-av-patientdata-samradkvb/*](https://vardgivare.skane.se/kompetens-utveckling/forskning-inom-region-skane/utlamnande-av-patientdata-samradkvb/)*; “Samrådsgrupp för kvalitetsregister, vårddatabaser och beredning”; so called KVB-group) or from the corresponding unit in Region Östergötland, Sweden – according to the “Law for ethical review of research on humans” [“Lag (2003:460) om etikprövning av forskning som avser människor”]. Thus, there are both ethical and legal restrictions on sharing even de-identified data due to potential identification as well as including sensitive information of the interviewed individuals. These restrictions are imposed by the mentioned Swedish Ethical Review Authority (see above). The data set consists of transcribed interviews which are not allowed to be presented publicly as decided in the approval by the Ethical Review Authority. “*

**Question:** Is the manuscript presented in an intelligible fashion and written in standard English?

PLOS ONE does not copyedit accepted manuscripts, so the language in submitted articles must be clear, correct, and unambiguous. Any typographical or grammatical errors should be corrected at revision, so please note any specific errors here.

Reviewer #1: Yes

Reviewer #2: Yes

**Reply:** Thank you.

Reviewer #1:

**Question:** Overall, it is an interesting work discuss the management challenges for an uncommon condition.
Conclusion can be made more informative and needs to be further expanded to reflect the findings from the current study.

**Reply:** Changes have been made in conclusion.

Reviewer #2: Review report
Question: I have thoroughly reviewed your article on addressing a qualitative study that after ulnar nerve surgery. What are the differences between your study and '' Kathleen Joy Khu at all. Patients’ Perceptions of Carpal Tunnel and Ulnar Nerve Decompression Surgery ''?
<https://www.cambridge.org/core/journals/canadian-journal-of-neurological-sciences/article/patients-perceptions-of-carpal-tunnel-and-ulnar-nerve-decompression-surgery/FFF7A6C677FB8354C92E520437998615>

**Reply:** Thank you for highlighting this study, where patients with carpal tunnel syndrome or mainly ulnar nerve entrapment at the elbow were interviewed 6-24 months after surgery. We have added the reference in our Discussions at two locations and made a slight comparison; however, their study differ substantially to ours. They emphasize based on their interviews that “(1) most patients did not perceive their condition to be serious; (2) patients were satisfied with the overall surgical experience; (3) the outcome was more important to patients than the process (4) majority of patients had a realistic expectation of outcomes.” Furthermore, they concluded that their patients “had a positive experience with carpal tunnel and ulnar nerve decompression surgery, although their level of satisfaction was dependent on the surgical outcome.”. Our patients had solely undergone surgery of the ulnar nerve entrapment at the elbow and our focus was on pain problems, which makes it difficult to make a thorough comparison. Therefore, we have only made a limited comparison.

We do hope that these changes are sufficient and that you now can consider the manuscript suitable for publication.

Sincerely yours

Alice Giöstad	Ingela K. Carlsson	Lars B. Dahlin	Erika Nyman
