# Supplementary material for: Experience of living with chronic pain in conjunction with surgery for ulnar nerve entrapment at the elbow—A qualitative study
Source: PLoS One. 2024 Jun 28;19(6):e0306327. doi: 10.1371/journal.pone.0306327 (PMC11213347; doi:10.1371/journal.pone.0306327)
Supplement: Attachment — Submitted filename: RESPONSE TO REVIEWERS.docx [file pone.0306327.s003.docx]

RESPONSE TO REVIEWERS – Rebuttal letter

Dear Editor,

Thank you for your mail concerning our manuscript entitled ”Experience of living with chronic pain in conjunction with surgery for ulnar nerve entrapment at the elbow – a qualitative study”. We appreciate the comments made by the reviewers and we have performed the requested changes in the manuscript.

A marked-up copy of our manuscript that highlights the performed changes made to the original version is attached in the system (labeled as 'Revised Manuscript with Track Changes'. Furthermore, an unmarked version of our revised paper without tracked changes as uploaded in the system and labeled as 'Manuscript'.

**Questions:**

**1. Sample size calculation not done. Kindly explain.**

**Reply**: Sample size calculation is common in quantitative research and sample size is most often determined before the start of the data collection for quantitative research questions. However, qualitative research studies uses smaller sample sizes but provides information in greater depth. Instead of calculating sample size beforehand, the sampling continues until data saturation has been achieved. Data saturation is reached when no new analytical information arises anymore. Two references is added in the methods section to clarify this approach.

**2. Sample size is very small (10). Kindly justify.**

**Reply**: The sampling in this study was made deliberately with purposive sampling which is a common method in qualitative research. In purposive sampling the researchers selects the participants that will be most informative and provide different perspectives of the studied phenomenon. We reached data saturation after ten interviews which was possible due to the information richness in the data and the variety of the participants.

**3. Nothing is mentioned about validation of semi-structured interview guide. Kindly explain.**

**Reply**: The semi-structured interview guide was developed in several steps and with care by the authors. The format of a semi-structured interview was considered as an appropriate method for data collection for this research question. By retrieving and using previous knowledge we gained a comprehensive and adequate understanding of the phenomenon under study. After this we formulated the preliminary semi-structured interview guide that was field tested in a pilot-interview with a potential study participant. The pilot interview was analysed to ensure that the research questions were being covered and that the questions were understandable. This approach is common in qualitative research. A reference have been added to the methods section to clarify this approach.

We do hope that these changes are sufficient and that you now can consider the manuscript suitable for publication.

Sincerely yours

Alice Giöstad	Ingela K. Carlsson	Lars B. Dahlin	Erika Nyman
